# Sensory integration abilities for balance in glaucoma, a preliminary study

**DOI:** 10.1038/s41598-021-98518-3

**Published:** 2021-10-04

**Authors:** Caitlin O’Connell, Mark Redfern, Kevin C. Chan, Gadi Wollstein, Ian P. Conner, Rakié Cham

**Affiliations:** 1 Department of Bioengineering, Swanson School of Engineering, University of Pittsburgh, Schenley Pl., #304, 4420 Bayard St, Pittsburgh, PA 15213 USA; 2 NYU Langone Eye Center, Department of Ophthalmology, NYU School of Medicine, NYU Langone Health, New York University, New York, NY USA; 3 Department of Radiology, NYU School of Medicine, NYU Langone Health, New York University, New York, NY USA; 4 Department of Ophthalmology, University of Pittsburgh, Pittsburgh, PA USA; 5 Department of Physical Therapy, University of Pittsburgh, Pittsburgh, PA USA

**Keywords:** Optic nerve diseases, Vision disorders

## Abstract

The goal of this study was to quantify the association between sensory integration abilities relevant for standing balance and disease stage in glaucoma. The disease stage was assessed using both functional (visual field deficit) and structural (retinal nerve fiber layer thickness) deficits in the better and worse eye. Balance was assessed using an adapted version of the well-established Sensory Organization Test (SOT). Eleven subjects diagnosed with mild to moderate glaucoma stood for 3 min in 6 sensory challenging postural conditions. Balance was assessed using sway magnitude and sway speed computed based on center-of-pressure data. Mixed linear regression analyses were used to investigate the associations between glaucoma severity and balance measures. Findings revealed that the visual field deficit severity in the better eye was associated with increased standing sway speed. This finding was confirmed in eyes open and closed conditions. Balance was not affected by the extent of the visual field deficit in the worse eye. Similarly, structural damage in either eye was not associated with the balance measures. In summary, this study found that postural control performance was associated with visual field deficit severity. The fact that this was found during eyes closed as well suggests that reduced postural control in glaucoma is not entirely attributed to impaired peripheral visual inputs. A larger study is needed to further investigate potential interactions between visual changes and central processing changes contributing to reduced balance function and increased incidence of falls in adults with glaucoma.

## Introduction

Glaucoma, an ocular condition characterized by a gradual loss of retinal ganglion cells leading to visual field deficits, is among the leading causes of low vision and blindness worldwide^1^. Individuals with glaucoma are at an increased risk of falling^2–4^. Falls impact mental and behavioral health. For example, fear of falling, activity restriction and physical deconditioning are ranked among the top health-related concerns in adults with glaucoma^5–8^. Prevalence estimates of glaucoma are age-dependent, ranging in the United States from about 0.4% in adults younger than 45 years old to over 10% in adults over the age of 75 years old^9^. It is expected that the prevalence of glaucoma will increase with the aging of the population^1,10^. Understanding why older adults with glaucoma fall more often than their healthy counterparts is critical to develop effective falls prevention and rehabilitation programs with appropriate intervention measures.

While glaucoma induced reduction in contrast sensitivity and the associated visual field loss have both been reported to be related to falls and vision-related disability^11,12^, the underlying mechanisms of falls are not well understood. Reduced vision is one possible mechanism of falls where individuals with glaucoma are less likely to detect environmental hazards compared to their healthy counterparts. Another potential mechanism is reduced postural control resulting in an impaired ability to centrally integrate sensory information relevant for balance. Sensory integration for postural control refers to the process of determining the position and motion of the body from three main sensory systems: vision, vestibular, and somatosensation^13^. When sensory cues from one system are absent or unreliable, healthy adults are able to effectively rely on the contributions of the other sensory systems in order to maintain balance, assuming the central processes relevant for balance are intact. For example, when visual inputs are unreliable for use by the postural control system, a greater reliance on somatosensory and vestibular inputs is required for equilibrium. This process is referred to as multisensory re-weighting or integration^13,14^. While impaired sensory integration abilities relevant for balance have not systematically been investigated in patients with glaucoma, several studies have reported worse balance when standing on foam, altering somatosensory information, in adults with glaucoma compared to controls^15–17^. These findings hint that balance impairments may be related to sensory integration deficits.

Thus, the goal of this preliminary study is to systematically assess balance-related sensory integration abilities in glaucoma as a function of disease stage. A well-established balance testing paradigm, involving dynamic posturography and specifically designed and validated for assessing sensory integration abilities^18^.

## Methods

The study was approved by The University of Pittsburgh Institutional Review Board. All methods were performed following the ethical principles stated in the Belmont Report, a requirement of The University of Pittsburgh Institutional Review Board. Written informed consent, approved by the University of Pittsburgh Institutional Review Board, was obtained before participation. Subjects diagnosed with glaucoma underwent an established assessment of their balance, focused on their abilities to centrally integrate sensory information relevant for postural control.

### Participants

Eleven individuals diagnosed with glaucoma were recruited for this study (Table 1). All recruited participants were clinically diagnosed with glaucoma after undergoing a comprehensive ophthalmic evaluation at the UPMC Eye Center that included a clinical exam, visual field testing (Humphrey Field Analyzer; Zeiss, Dublin, CA), and a spectral-domain optical coherence tomography (Cirrus HD-OCT, Zeiss, Dublin, CA). Participants were able to stand for at least 2 h. Exclusionary criteria were self-reported diabetes, orthopedic, neurological, pulmonary, or cardiovascular conditions that may negatively impact balance and ocular pathologies other than glaucoma. Potential participants were also excluded if (1) they were taking any central nervous system anti-depressant drugs, including benzodiazepines or barbiturates, or taking more than five prescription drugs, as both may increase fall risk^19,20^, (2) they had reduced proprioceptive or plantar cutaneous sensory function based on established age-related norms^21–24^; and with (3) a self-reported history of vertigo. Finally, all participants reported a negative 12-month falls history.

**Table 1:** Characteristics of participants with glaucoma.

Subject ID	Gender	Age (years)	VF MD better eye (dB)		VF MD worse eye (dB)	RNFL thickness better eye (μm)	RNFL thickness worse eye (μm)	Glaucoma diagnosis
1	F	74	− 10.28		− 10.52	60	57	Chronic angle closure
2	M	65	− 8.57		− 12.00	74	65	Primary open angle
3	F	80	− 4.89		− 21.04	75	57	Normal tension
4	F	56	− 2.07		− 3.94	74	73	Open angle
5	M	66	− 1.80		− 3.47	66	60	Primary open angle
6	M	70	− 1.36		− 1.96	73	63	Primary open angle
7	F	54	− 1.10		− 1.73	85	75	Pigment dispersion
8	F	54	− 0.27		− 0.30	98	95	Primary open angle
9	M	72	− 0.20		− 14.94	77	61	Pseudoexfoliation
10	F	69	0.46		− 2.01	63	60	Low tension
11	F	60	0.69		− 3.30	89	71	Low tension
12	M	56	0.99		− 17.57	104	76	Primary angle closure
Mean ± S.D		65 ± 9	− 2.4 ± 3.7	− 7.7 ± 7.1		78 ± 13	68 ± 11	

Glaucoma severity was determined in two ways: (1) using a functional measure, specifically visual field mean deviation (VF MD) assessed by automated Humphrey perimetry and quantifying visual field deficits, and (2) using a structural measure, specifically retinal nerve fiber layer (RNFL) thickness as measured by OCT. Both measures have been used to classify disease stage. Specifically, Hodapp and colleagues established a VF MD- based classification^25^, with early, defined as VF MD greater than − 6 dB, moderate, defined as VF MD ranging between − 12 and − 6 dB, and advanced, defined as VF MD less than − 12 dB. VF MD in the better eye has been related to functional mobility impairments. While early RNFL thinning are not associated with visual disability, it often precedes functional loss and is thus an important variable that may be the first sign of glaucomatous damage^26^. In addition, RNFL thinning has also been linked with reduced cognitive function^27^ and the presence of dementia^28,29^. Given that the integrity of higher cognitive functioning may hinder sensory integration processes relevant for balance^30–34^, RNFL thickness was considered as another assessment of disease stage.

There is also a debate in the literature related to whether disease stage should be assessed using vision data from the worse eye or better eye. While disease stage in the better eye has traditionally been used as a clinical assessment of visual function and quality of life^35,36^, the most affected eye or worse eye has been implicated in structural changes in the brain^26^, cognitive impairments^37^ and even quality of life measures^38^. In this study, while it is anticipated that data from the better eye will be most relevant, data from both eyes were considered.

### Protocol

The standing balance test used dynamic posturography on an Equitest posture platform (Neurocom, Inc) located in the Jordan Balance Disorders Laboratory within the Eye & Ear Institute of Pittsburgh. The Equitest platform is capable of sway-referencing the floor and/or visual environment, which provides rotations of the supporting floor and/or visual scene in direct proportion to an individual’s sway magnitude in the anterior–posterior direction. Sway-referencing the floor causes movements of the supporting surface in an attempt to keep the ankle angle constant, thus reducing and altering somatosensory information from the ankle and requiring the person to rely on vision and vestibular inputs for balance^39^. Similarly, sway-referencing the visual scene in proportion to the individual’s sway will reduce balance-related visual cues. The platform records ground reaction forces under the feet during standing and the underfoot center of pressure (COP) is computed and saved during the trials. Participants wore a safety harness that would prevent hitting the floor in the event of a balance loss. During balance testing, participants were instructed to stand as still as possible without locking their knees. Participants were assessed in 6 postural conditions (Table 2), each lasting 3 min (an adapted version of the Sensory Organization Test, a well-established balance testing paradigm used and validated in healthy and clinical populations^18^). These sensory challenging conditions alter and/or reduce sensory information relevant for balance in a systematic manner (Table 2).

**Table 2:**
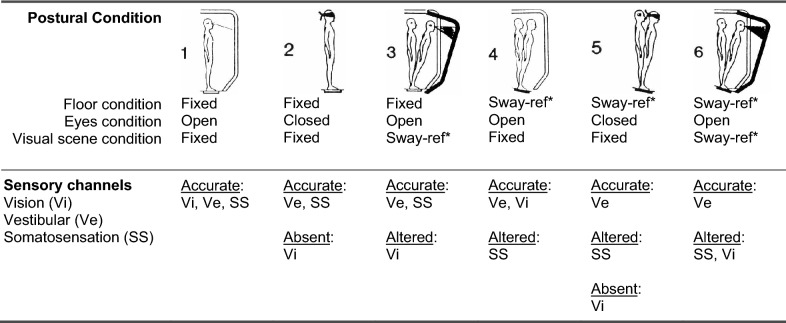
Postural conditions included in the balance assessment, an adapted version of the Sensory Organization Test Conditions^18^.

*Sway-referenced.

### Data Processing and Analyses

The COP data were low-pass filtered using a fourth-order Butterworth filter with a cutoff frequency of 2.5 Hz, and down-sampled to 20 Hz. The first 30 and last 5 s of the COP time series were removed to eliminate any transient effects and thus allowing the adaptation to a new postural condition to occur. Anterior–posterior COP data were used to quantify postural sway since sway-referencing was in the anteroposterior direction. Sway magnitude was assessed using the root-mean-square of the filtered COP displacement (COP RMS). The speed of movement was assessed by using the time-normalized path length of the COP data (COP NPL), calculated by summing the absolute value of the differences over time.

Preliminary analyses were focused on investigating the impact of the *postural condition* (PC1, …, PC6) on sway measures (COP RMS and COP NPL) using a mixed linear model with the postural condition as the fixed effect and subject as the random effect. To examine the association between glaucoma severity and balance in the main analyses, mixed linear models were used in JMP Version 10 (SAS Institute Inc), with the fixed effects including a glaucoma severity measure (one of four possible measures as explained below), postural condition (PC1, …, PC6) and the interaction of these factors. Subject was also added as a random effect. Four measures of glaucoma severity were individually considered as independent measures in these statistical models: (1) functional measure of glaucoma severity (VF MD) of the better and worse eye, and (2) structural measure of glaucoma severity (RNFL thickness) of the better and worse eye. The dependent variables of interest in both the preliminary and main analyses were the log-transformation of the sway measures, specifically COP RMS and COP NPL. Both of these measures were log-transformed to satisfy the required normality assumption. Statistical significance was set at 0.05.

## Results

*Preliminary analyses: postural condition influence on sway measures.* As anticipated, preliminary analyses revealed a statistically significant impact of the *postural condition* (PC1, …, PC6) on both sway magnitude, i.e. COP RMS (F(5,54) = 136.1, p < 0.0001, Table 3), and sway speed, i.e. COP NPL (F(5,54) = 62.0, p < 0.0001, Table 3). More specifically, post-hoc Tukey comparison tests indicated that sway-referencing the floor caused the greatest increase in sway magnitude while closing the eyes or sway-referencing the visual environments caused minimal changes in sway magnitude. In other words, sway magnitude was significantly larger in postural conditions (PC4 … PC6) than in (PC1 … PC3), but no statistically significant differences were found between PC1, PC2, and PC3 and between PC4, PC5, and PC6 (Table 3). Similar results were found when post-hoc Tukey tests were used to contrast sway speed between postural conditions (Table 3), i.e. subjects swayed faster in postural conditions (PC4, …, PC6) than in conditions (PC1, …, PC3). In addition, faster sway speeds were noted in closed-eyes conditions (PC5, PC2) compared to open eyes, fixed environments (PC4, PC1) conditions, respectively.

**Table 3:** Mean (standard deviation) of sway magnitude (COP RMS) and sway speed (COP NPL).

	COP RMS (cm)	COP NPL (cm/s)
PC1: fixed floor, eyes open, and fixed visual scene	0.57 (0.19)	0.73 (0.20)
PC2: fixed floor, eyes closed	0.69 (0.19)	0.96 (0.25)
PC3: fixed floor, eyes open and sway-referenced visual scene	0.65 (0.16)	0.90 (0.29)
PC4: sway-referenced floor, eyes open, and fixed visual scene	1.62 (0.65)	2.32 (1.09)
PC5: sway referenced floor, eyes closed	1.77 (0.54)	3.03 (0.96)
PC6: sway referenced floor, eyes open and sway-referenced visual scene	2.05 (0.46)	2.70 (0.71)

**Figure 1:**
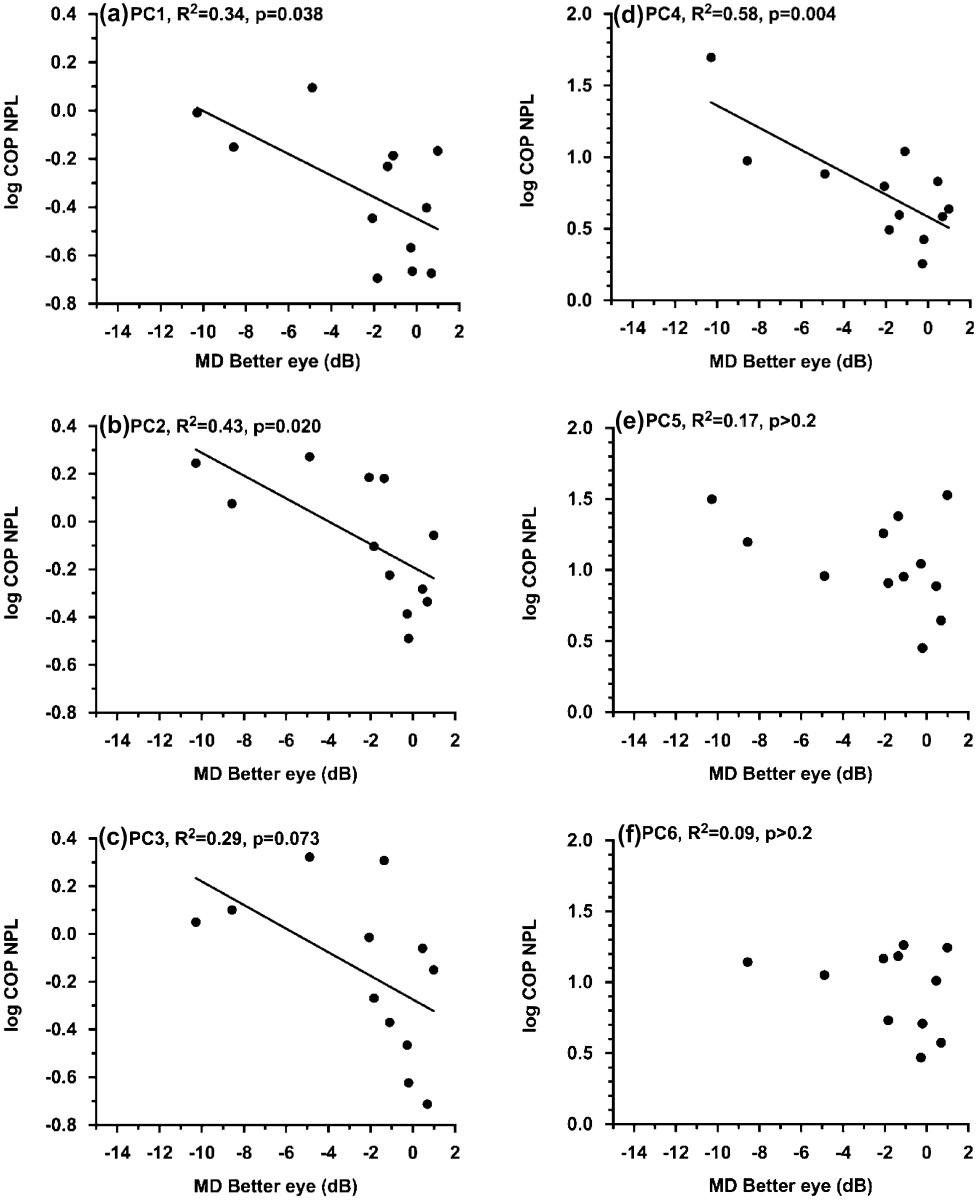
Association between visual field in the better eye (MD better eye) and normalized path length (COP NPL) when exposed to the 6 postural conditions (PC1, …, PC6). A more negative visual field median deviation (VF MD) value on the x-axis indicates worse visual field deficits. Note a significant correlation indicated by the line between COP NPL and MD better eye.

*Main analyses: influence of glaucoma severity on sway.* The analyses using the visual field deficit in the better eye (MD better eye) as the glaucoma severity measure revealed a statistically significant MD better eye-related effect on sway speed (COP NPL) (F(1,10) = 8.1; p = 0.017). More specifically, COP NPL increased with a greater visual field deficit in the better eye (Fig. 1). While this effect was in general consistent across postural conditions (i.e. effect of *MD better eye x postural condition* interaction on COP NL was not statistically significant, F(5,49) = 0.93, p = 0.47), it was less prominent in postural conditions PC5 and PC6 (Fig. 1e,f). In contrast to the findings related to the visual field deficit in the better eye, the effect of the visual field deficit in the worse eye on sway speed did not reach statistical significance (F(1,10) = 1.1; p = 0.32). Also, there was no statistically significant effect of visual field deficits in either eye on sway magnitude (COP RMS) (p > 0.5). Finally, the analyses using RNFL thickness as a measure of glaucoma severity revealed that structural damage in either eye did not impact sway (COP NPL and COP RMS, p > 0.3).

## Discussion

The main result of this study is that worse visual field deficit in the better eye was associated with increased speed of sway under four of the six postural conditions. Visual field deficits in the worse eye and structural damage in either eye were not associated with balance measures under the postural conditions. In addition, similarly to other older populations, reducing somatosensory information on a sway-referenced platform (PC4-PC6) in adults with glaucoma increases postural instability and challenges the postural control system.

The underlying mechanism that results in associations between glaucoma and postural control is not clear. The finding that visual deficits in the better eye are associated with increased sway speed suggests that poorer overall visual fields lead to reduced postural control. However, note that a strong association was seen not only when vision was available, but also during the eyes closed condition (PC2). This may suggest that reduced postural control in glaucoma is not entirely attributed to impaired peripheral visual inputs, but may also be partially attributed to a central sensory integration mechanism. Supporting this hypothesis are the findings of neuroimaging studies that suggest widespread brain structural and functional alterations in glaucoma^26,40–44^, including in areas that may be involved in sensory integration processes relevant for balance^41^. In these imaging studies, the reported brain changes are dependent on the disease severity and are more prominent with worse visual field deficits^40,42,45^. Thus, associated changes in the brain with worsening visual field deficits may be a partially mediating factor in postural control seen in this study.

It is worthwhile noting that only sway speed (COP NPL), not sway magnitude (COP RMS), was associated with glaucoma severity. Sway magnitude reflects the output of the postural control system, i.e. COP RMS reflects how well balance is maintained. In contrast, COP NPL, a measure of sway speed, reflects, at least partially, the challenges experienced by the postural control system to identify and to generate an appropriate balance response^46^.

Prior studies have shown altered balance in adults with glaucoma when standing on foam^15–17,47^ is in general consistent with our findings using dynamic posturography to assess balance. Sway in patients with glaucoma is increased in general. The only study to examine both functional and structural measures of glaucoma and their relationship with balance found RNFL thickness to be a better predictor of visual contribution to balance than VF MD^15^. It is difficult to compare the results of studies with foam and dynamic computerized posturography. Fundamental differences between using foam versus sway-referencing the flooring surface are^48^: (1) the balance test in this study uses a non-compliant supporting surface that is computer-controlled to move in phase with an individual’s sway in the anterior–posterior direction, keeping the ankle angle at 90°. Thus, sway-referencing the floor, the method used in this study, is an effective way to minimize balance-related somatosensory cues at the ankles and to induce instability mainly in the anterior–posterior direction; (2) in contrast, a foam surface is compliant in all directions, and thus induces instability in multiple directions by altering (i.e. introducing noisy) sensory information at the receptors located on the bottom of the feet. Postural control studies have indeed shown those balance assessments using sway-referenced floors and foam surfaces are not always correlated^48–50^.

Limitations to the present study are the relatively small sample size and the limited spread of glaucoma severity. However, the fact that statistically significant effects were detected with this sample size at these severity levels suggests further studies in a larger patient population are needed. Another potential limitation was that the impact of the location of glaucomatous damage in the visual field was not examined. Studies have suggested that inferior visual field loss negatively impacts balance and mobility to a greater extent than the loss in the superior visual field^2,51,52^. However, there are some inconsistencies in literature as de Luna et al. did not find any significant difference in sway measures between glaucoma patients with superior versus inferior visual field loss in a large participant group^47^. Future work will need to consider the impact of location of visual field loss. Finally, the heterogeneity of the research participants in general is another factor to consider. A diverse group (e.g., glaucoma diagnosis, unilateral versus bilateral vision loss, etc.) of participants was recruited. While this factor may be considered a weakness, this study still allowed to establish the association between glaucoma severity and sensory integration abilities relevant for balance even in this heterogeneous group of research participants. Future research can now further investigate this association more specifically in more homogeneous group.

In summary, this study found that balance is impacted by glaucoma under conditions where sensory integration is challenged. The finding that visual field severity and sway speed are associated during the eyes-closed condition suggests a central sensory integration mechanism, consistent with recent findings that glaucoma impacts brain regions involved in balance control. Further research is warranted.
